# Experiences of menstrual health in the Nordic countries: a scoping review of qualitative research, applying an intersectional lens

**DOI:** 10.1080/26410397.2024.2446081

**Published:** 2025-02-14

**Authors:** Eva Åkerman, Anna Wängborg, Maria Persson, Marie Klingberg-Allvin

**Affiliations:** a Postdoctoral Researcher, Department of Women’s and Children’s Health, Karolinska Institutet, Stockholm, Sweden.; b PhD student, Department of Women’s and Children’s Health, Karolinska Institutet, Stockholm, Sweden; c PhD student, Department of Public Health Sciences, Stockholm University, Stockholm, Sweden; d Professor, Department of Women’s and Children’s Health, Karolinska Institutet, Stockholm, Sweden

**Keywords:** Menstrual health, menstrual cycle, experience, intersectionality, Nordic countries

## Abstract

Achieving menstrual health is fundamental to gender equality, human rights, and the well-being of all people who menstruate. We undertook a scoping review to map the extent and range of qualitative studies on menstrual health in the Nordic countries and applied an intersectional lens in reporting the findings. The specific research questions we aimed to answer were (1) what types of menstrual health experiences were researched, (2) whose experiences and voices were being researched, and (3) what gaps exist in understanding the experiences and challenges encountered by diverse groups. Four databases were searched for peer-reviewed articles published between 2011 and 2023. Searches yielded 2733, and 22 articles met our inclusion criteria. Included studies were undertaken in Denmark (*n* = 5), Iceland (*n* = 1), Norway (*n* = 3), and Sweden (*n* = 13). The samples included menstruating people, healthcare professionals, and/or other professionals. Most of the included studies reported on menstrual experiences related to menstrual pain and disorders such as endometriosis. Studies focusing on understanding menstrual health experiences among people in vulnerable situations in the Nordic countries are lacking. We found that menstrual health experiences of menstruating people with the following identities were under-researched: people with disabilities, non-Nordic ethnicities, refugees, gender-diverse people, people experiencing homelessness, and young adolescents. The findings suggest that we have little knowledge and understanding of the experiences and challenges that might be faced by these groups in the Nordic countries. Findings of this scoping review can be used to inform future research directions and policy programming.

## Plain language summary

Menstrual health is a gender and human rights issue. It is fundamental to all people who menstruate. We reviewed evidence on menstrual health in the Nordic countries to find research gaps. We aimed to answer three research questions. (i) What types of menstrual health experiences have been studied? (ii) Whose experiences and voices are being studied or left out? (iii) What gaps exist in understanding the experiences and challenges faced by different groups of people? We searched four databases for articles published between 2011 and 2023. We screened 2733 articles and included 22. They were from Denmark (*n* = 5), Iceland (*n* = 1), Norway (*n* = 3) and Sweden (*n* = 13). These studies included experiences of menstruating individuals, healthcare professionals, and other professionals. Most studies focused on experiences related to menstrual pain and disorders like endometriosis. We found a lack of research on menstrual health of: people with disabilities; non-Nordic ethnic backgrounds; refugees; gender-diverse people; people experiencing homelessness; and young adolescents. This leaves us with little understanding of their menstrual health experiences and challenges. Our review can inform future research and policy.

## Résumé

Parvenir à la santé menstruelle est fondamental pour l'égalité des genres, les droits humains et le bien-être de toutes les personnes qui ont des menstruations. Nous avons entrepris une étude de portée pour recenser l'étendue et la diversité des études qualitatives sur la santé menstruelle dans les pays nordiques et nous avons appliqué une approche intersectionnelle dans la présentation des résultats. Les questions de recherche spécifiques auxquelles nous souhaitions répondre étaient : 1) quels types d'expériences en matière de santé menstruelle ont été étudiés ; 2) à qui appartenaient les expériences et les voix ayant fait l'objet des recherches ; et 3) quelles sont les lacunes dans la compréhension des expériences et des défis rencontrés par divers groupes. Nous avons cherché des articles de revues à comité de lecture publiés entre 2011 et 2023 dans quatre bases de données. Ces recherches ont produit 2733 articles dont 22 correspondaient à nos critères d'inclusion. Les études retenues avaient été entreprises au Danemark (n=5), en Islande (n=1), en Norvège (n=3) et en Suède (n=13). Les échantillons comprenaient des personnes ayant des menstruations, des professionnels de santé et/ou d'autres professionnels. La plupart des études incluses relataient des expériences menstruelles liées aux douleurs et troubles menstruels comme l'endométriose. Les études axées sur la compréhension des expériences en matière de santé menstruelle chez les personnes en situation de vulnérabilité dans les pays nordiques font défaut. Nous avons constaté que les expériences de santé menstruelle des personnes menstruées ayant les identités suivantes étaient sous-étudiées : personnes handicapées, ethnies non nordiques, réfugiés, personnes de divers genres, sans-abris et jeunes adolescents. Les résultats suggèrent que nous connaissons et comprenons mal les expériences et les défis que ces groupes peuvent rencontrer dans les pays nordiques. Les conclusions de cette étude de portée peuvent être utiles pour guider les futures orientations de la recherche et la programmation des politiques.

## Resumen

La salud menstrual es fundamental para la igualdad de género, los derechos humanos y el bienestar de todas las personas menstruantes. Realizamos una revisión de alcance para mapear la extensión y el alcance de los estudios cualitativos sobre la salud menstrual en los países nórdicos y aplicamos una perspectiva interseccional para reportar los hallazgos. Nos propusimos contestar las siguientes preguntas específicas de investigación: 1) qué tipos de experiencias de salud menstrual han sido investigadas, 2) de quiénes son las experiencias y voces investigadas y 3) qué lagunas existen para entender las experiencias y los retos enfrentados por diversos grupos. En cuatro bases de datos, se hizo una búsqueda de artículos revisados por pares publicados entre 2011 y 2023. La búsqueda produjo 2733 artículos y 22 de ellos reunían nuestros criterios de inclusión. Los estudios incluidos fueron realizados en Dinamarca (n = 5), Islandia (n = 1), Noruega (n = 3) y Suecia (n = 13). Las muestras incluyeron a personas menstruantes, profesionales de salud y/u otros profesionales. La mayoría de los estudios incluidos informaron sobre experiencias menstruales relacionadas con dolor y trastornos menstruales, tales como la endometriosis. Se carece de estudios centrados en entender las experiencias de salud menstrual de personas en situaciones vulnerables en los países nórdicos. Determinamos que no se han realizado suficientes investigaciones sobre las experiencias de salud menstrual de personas menstruantes con las siguientes identidades: personas con discapacidad, etnias no nórdicas, refugiadas, personas con diversos géneros, personas sin vivienda y adolescentes jóvenes. Los hallazgos indican que tenemos poco conocimiento y comprensión de las experiencias y los retos que podrían enfrentar estos grupos en los países nórdicos. Los hallazgos de esta revisión de alcance pueden utilizarse para incidir en nuevas futuras direcciones de investigación y de programación de políticas.

## Introduction

More than a quarter of the world’s population is of reproductive age and most of them menstruate for about two to seven days monthly.^1^ Menstruation has historically been under-researched^2^ but there is increasing recognition of its importance in achieving the sustainable development goals, and realising gender equality and human rights.^3,4^ Menstrual health is defined as “a state of complete physical, mental, and social well-being and not merely the absence of disease or infirmity, in relation to the menstrual cycle”.^5^ To achieve menstrual health, the definition establishes five requirements: (i) access to accurate information and education about the menstrual cycle throughout the lifespan, (ii) access to materials, facilities, and services that support personal preferences, hygiene, comfort, privacy, and safety in caring for the body, (iii) access to timely diagnosis, treatment, and care for menstrual cycle-related discomfort and disorders, (iv) experiencing a positive and supportive social environment free from stigma and psychological distress, and (v) freedom to participate in all spheres of life throughout the menstrual cycle, free from menstrual-related exclusion, restriction, and discrimination. The definition of menstrual health is holistic and acknowledges the nuanced understanding of menstrual experiences and their intersections with physical, mental, and social health. In this review, our understanding of menstrual health is based on this holistic definition. Although the majority of those who experience a menstrual cycle are women or girls, it should be acknowledged that not all women and girls menstruate and not all menstruating people define themselves as “girls or women”. In this review, we will use various terms such as girl, woman, and menstruating person/people when referring to people who experience a menstrual cycle. Furthermore, we have chosen to retain the terms used in the included studies.

Menstrual-related disorders such as menstrual pain, heavy bleeding, endometriosis, premenstrual syndrome (PMS), and premenstrual dysphoric disorder can have a negative impact on quality of life for people who menstruate^6–11^ and affect their participation in social activities, education, and work.^7,9,11–13^ Menstrual disorders are common; for instance, menstrual pain affects around 70–90% of young women worldwide.^14^ Despite symptoms, many people who menstruate refrain from seeking medical care due to a normalisation of symptoms such as pain and heavy bleeding,^15,16^ low levels of menstrual literacy/lack of access to information,^15,16^ and menstrual stigma and taboo.^17,18^ Menstrual stigma refers to negative attitudes towards menstruation or people who menstruate.^19^ In addition to limiting access to care, menstrual stigma also limits access to social support and information and leads to behavioural expectations which prohibit or prevent women, girls, and menstruating people from participating in education and work life.^12,20–22^

Previous reviews have focused on specific components of menstrual health or specific contexts, none focusing on the Nordic region. For example, research on menstrual health literacy and menstrual hygiene management has predominantly focused on low- and middle-income countries (LMICs),^15,23–25^ with comparatively less attention given to high-income countries (HICs) like those in Europe. In HICs, most reviews have focused on treatment, diagnosis, and tackling of menstrual disorders and pain,^26,27^ along with investigating their associations with adverse impacts on quality of life and employment.^17,28,29^ Menstrual health experiences are shaped by many factors, such as disability, age, gender identity, place of residence, homelessness, religion, ethnicity, caste and culture, and intersecting identity factors.^5^ To the best of our knowledge, no published reviews have focused on menstrual health experiences applying an intersectional lens.

The term “intersectional” was coined in 1989 by Kimberlé Crenshaw, an American civil rights advocate, and is rooted in Black feminist and critical legal theory.^30^ Although there is no shared definition of intersectionality and consensus on how to apply the theory, attention to social justice, power, and interacting dimensions of identities is common.^31,32^ Intersectionality asserts that collective social identities encompassing class/socio-economic status (SES), race/ethnicity, nationality, gender/gender identity, sexual orientation, and disabled status intersect in multiple interconnected structures of power resulting in differing experiences of privilege and oppression. Intersectionality is recognised as a useful and essential theoretical framework to address health concerns and to map health disparities with more precision in order to outline more effective directions in policy and programme development.^32^ In this study, an intersectionality lens was applied when analysing the included studies to understand whose voices were and were not being researched, and to map the menstrual health experiences and challenges encountered by groups with various intersecting identities. The results of this scoping review can be used to inform future health research programmes and policies.

### Aim and specific research questions

The aim of this scoping review was to map the extent and range of qualitative research on menstrual health in the Nordic countries, applying an intersectionality lens. The specific research questions we aimed to answer were:

- What types of menstrual health experiences and topics have been researched?
- Whose experiences and voices are/are not being researched, and what gaps exist in understanding the experiences and challenges encountered by diverse groups with different intersecting identities?

## Methods

This review was conducted following Arksey and O’Malley’s five-stage framework for scoping reviews^33^: identifying research questions, identifying relevant studies, selecting studies, charting data, and collating findings. The Preferred Reporting Items for Systematic Reviews and Meta-Analyses – Extension for Scoping Reviews (PRISMA-ScR) guideline and checklist were used in reporting.^34^ We did not register or publish our study protocol.

### Search strategy

A literature search was performed in the following databases: Medline, Web of Science, PsycInfo, and CINAHL. The original search was performed on 26 January 2022, after which the search was updated on 9 June 2023 using the methods described by Bramer et al.^35^ Before the final search strategy was developed, several pilot searches were conducted using terms identified in Medical Subject Headings (MeSH) and free-text terms.

The final search strategy was developed in collaboration with an information specialist at the Karolinska Institutet University Library. The initial search strategy was developed in Medline, where MeSH terms and free-text terms were identified for each search concept. These terms were then translated into the other databases using Polyglot Search Translator.^36^ The search strategy was subsequently adapted for databases Web of Science, PsycInfo, and CINAHL. This process involved not only translating the syntax, but also converting MeSH terms into their equivalents, such as CINAHL Headings and APA Thesaurus of Psychological Index Terms. No language restriction was applied.

### Eligibility criteria

Based on our research questions, we used the inclusion criteria shown in Table 1 to identify studies of interest. The inclusion and exclusion criteria were developed parallel to the pilot searches. At an early stage of our screenings, specific disorders, such as von Willebrand disease and eating disorders, were set as exclusion criteria from the study due to the complex ways these conditions can influence menstruation. Studies related to menopause were also set as exclusion criteria. Due to symptoms that occur in the later stage of reproductive years, we found this topic requires a separate focus for future review.

**Table 1.** Inclusion and exclusion criteria

	Inclusion criteria	Exclusion criteria
Study population	Girls, women, transgender, non-binary, and all people with experience of the menstrual cycle and professionals working with menstrual health	
Context	Nordic countries (Denmark, Finland, Island, Norway, and Sweden)	
Publication type	Primary studies, published in peer-reviewed journals	No full-text available, grey literature, reviews or secondary analysis, protocols, commentaries, debates, and validation studies
Year	January 2011–June 2023	
Study design	Qualitative design	Policy analysis. Quantitative design
Language	English, Danish, Finnish, Norwegian, Icelandic, and Swedish	
Menstrual health area/topic	Experiences of care, treatment, quality of life, well-being and lifestyle factors related to the menstrual cycle, as well as symptoms and problems such as dysmenorrhea (menstrual pain), menorrhagia (heavy bleeding), endometriosis, fibroids, polycystic ovary syndrome, premenstrual syndrome, premenstrual dysphoric disorder	Fertility research, pregnancy, stem cell research, miscarriage and bleeding, abortion and bleeding, and contraception as birth control, pre-menopause, perimenopause and menopause, research on animals, climacterium, prevalence of menstrual disorders, menstrual health among people with eating disorders, and von Willerbrands

The inclusion and exclusion criteria were refined throughout the process of identifying and selecting studies.

### Study selection

**Figure 1.**
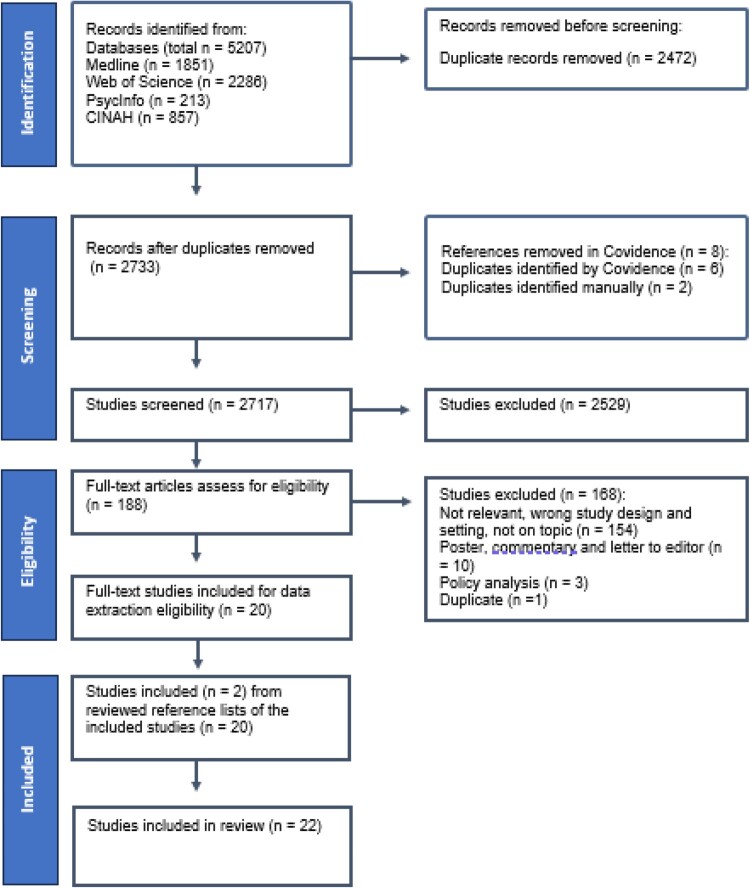
PRISMA flowchart of the screening and study inclusion process

The study selection was conducted by two researchers (AW and EÅ) independently and was guided by the research questions and the inclusion and exclusion criteria (Table 1). The identified articles were imported to Covidence, a web-based programme to facilitate screening in literature reviews. A two-step process was applied in both the original search and the updated search (Figure 1). In total, 2717 titles and abstracts were screened for relevance by two researchers (AW and EÅ) independently. This was followed by 188 full-text screenings. Disagreements were resolved through discussion, with the aim and research questions taken into consideration. Hand searching of reference lists was performed by one researcher (EÅ).

### Data charting

A data extraction form in Microsoft Excel was used to chart author names, article title, journal discipline, country, year, sample sizes, study aim, context, type of menstrual experience, and participant characteristics (Table 2, Table 3). The results text was extracted from full-text and can be found in Supplementary Table A1. A data extraction form was also used to identify characteristics covering intersecting identities such as age, disability, ethnicity/race, gender, gender identity, sexual identity, SES, and religion (Table 4, Supplementary Table A2). Data extraction was conducted by two researchers (AW and EÅ) who cross-checked each other's work. A narrative approach applying an intersectionality lens was used to summarise the findings of the included studies. All authors reviewed and discussed the findings. The use of intersectionality in analysing whose experience was being researched, allowed us to identify whose voices and experiences had been left out from menstrual health research in the Nordic countries.

**Table 2.** Distribution of when and where studies were published and conducted, and methods used

Publication characteristics	Distribution *n* = 22
**Year of publication**	
2021–2023	9
2018–2020	6
2015–2017	4
2012–2014	3
**Journal discipline**	
Health care (sexual and reproductive health care)	5
Healthcare nursing research	3
Health care	3
Culture, health and sexuality	2
Caring science	2
Complementary and alternative research	1
Healthcare nursing paediatric	1
Health and well-being	1
Environmental research and public health	1
Media research	1
Sexuality and disabilities	1
Sport science	1
**Methods used**	
Individual interviews	16
Individual interviews and focus group discussions	1
Semi-structured interviews and participant observations	1
Participant observations	1
Mixed method including questionnaire, individual interviews and focus group discussions	1
Mixed method including questionnaire and focus group discussions	1
Analysing blog posts	1
**Country**	
Sweden	13
Denmark	5
Norway	3
Iceland	1

**Table 3.** Types of menstrual health experiences and study aims

Characteristics of population and sample size (*n*)	Specific context and country	Types of menstrual health experiences	Study aim	Reference
Seven women aged 12–53 years diagnosed with endometriosis. *n* = 7	Endometriosis patient association, Facebook groups for women with endometriosis. Denmark	Endometriosis	To explore the lived experiences of endometriosis in adolescence and the social reactions impact on the illness experience and quality of life	^42^
Sixteen written blogs by women aged 22–34 years diagnosed with endometriosis. *n* = 16	Blogs written by people diagnosed with endometriosis. Sweden	Endometriosis	To identify and describe endometriosis healthcare experiences based on affected individuals’ blog posts.	^45^
Twelve women aged 18–45 years with endometriosis. *n* = 12	Online support forum focusing on healing and diet. Sweden	Endometriosis	To explore how persons with endometriosis experienced their health after dietary changes	^44^
Thirteen women aged 24–48 years diagnosed with endometriosis participating in treatment for chronic pain at the clinic. *n* = 13	Specialised pain clinic. Sweden	Endometriosis	To examine women’s experience of painful endometriosis including long-term aspects, social consequences, impact of treatment and development of own coping strategies	^41^
Ten women aged 20–45 years with endometriosis. *n* = 10	Gynaecological outpatient clinic. Denmark	Endometriosis	To assess changes in self-reported endometriosis-specific health-related quality of life (HRQOL) and confidence in managing health and care	^43^
Nine women aged 23–55 years with endometriosis. *n* = 9	University hospital and central hospitals. Sweden	Endometriosis	To identify and describe the experience of healthcare encounters among women with endometriosis	^40^
Twelve women with experience of menstruating aged 18–48 years. *n* = 12	Non-specific context. Sweden	Menstruation	To describe women’s experiences of menstruation across the lifespan	^50^
Thirteen female elite cross-country skiers aged 20–35 years and eight of their coaches aged 30–60 years (two women and six men). *n* = 21	Sports context, cross-country skiers at national level. Sweden	Menstrual cycle communication	To gain an in-depth understanding of the perceptions and experiences of elite female endurance athletes and their coaches in relation to barriers to communication about MC and HC issues	^48^
Eight female junior elite footballers aged 20–35 years and two of their male coaches (age was not reported). *n* = 10	Sports context, elite footballers at highest junior level. Norway	Menstrual cycle communication	To explore the perceptions of MC communication in a group of junior elite football players and their male coaches in a case study of one youth football team in a specific club in Norway	^49^
Nine young women aged 16–22 years originating from Somalia who reported being infibulated *n* = 9	Gynaecological clinic, youth clinic and health facility. Sweden	Menstrual pain in relation to infibulation	To explore the lived experiences of young migrant women from Somalia and their views on undergoing medical defibulation in Sweden	^51^
Eight women aged 19–46 years originating from Ethiopia, Somalia, Djibouti, and Eritrea. *n* = 8	Non-specific context. Sweden	Menstrual pain in relation to infibulation	To explore how women from part of the world where FGM is normative perceive and experience FGM after immigrating to Sweden	^52^
Twenty-three women and 23 men aged 18–65 years originating from Somalia and Sudan *n* = 23 (men excluded as they did not report experiences regarding menstrual health)	Non-specific context. Norway	Menstrual pain in relation to infibulation	To explore experiences and perceptions of premarital defibulation	^53^
Twenty-five people with intellectual disabilities who require intensive support and communicate with non-spoken language. Twelve women and 13 men aged 26–66 years. *n* = 12 (men excluded as they did not reported experiences regarding menstrual health)	Group homes and flats, assisted living arrangements. Iceland	Menstruation, blood and contraception	To demonstrate how the sexuality of people with intellectual disabilities who require intensive support is shaped by sociocultural sexual scripts and the support they receive in everyday life	^55^
Eight women aged 23–38 years with diagnosis of PCOS. *n* = 8	Women with PCOS included in an earlier randomised controlled trial. Sweden	PCOS, acupuncture treatment for women diagnosed with PCOS	To describe the experience of acupuncture for women diagnosed with PCOS	^46^
Twenty-one women aged 21–36 years with PCOS. *n* = 21	Women with PCOS who had signed up to participate in physiological research project. Denmark	PCOS suffering with several symptoms: hirsutism, fertility problems and irregular/lack of menstrual cycle	To explore women’s lived experiences of PCOS	^46^
Twelve women aged 26–40 years who use period trackers. *n* = 12	Utilisation of digital platform. Denmark	Period trackers	To explore menstrual stigma and the usage of period trackers and investigate how digital traces from datafied bodies transmit meaning in everyday life	^54^
Seven midwives and six gynaecologists aged 45–65 years caring for defibulated immigrant women. *n* = 13	Health care facilities. Sweden	Menstruation in relation to defibulation	To capture care providers’ perceptions of defibulated immigrant women’s sexual and reproductive health, illuminated by their experiences as care providers for such women	^59^
Fifteen school nurses aged 36–58 years (14 female and 1 male). *n* = 15	Schools. Sweden	Menstrual pain	To describe school nurses’ experiences of supporting girls with menstrual pain	^58^
Fifteen female midwives aged 28–63 years with of supporting girls with menstrual pain. *n* = 15	Youth clinics. Sweden	Menstrual pain among youth	To describe midwives’ experiences of supporting girls with menstrual pain	^57^
Nine female gynaecological nurses aged 24–61 years who were part of an endometriosis team. *n* = 9	Gynaecological units in an urban hospital. Denmark	Women with endometriosis	To explore how the personal attitudes of gynaecological nurses, their specialised knowledge and their clinical experiences influenced the way they conceptualised and cared for women with endometriosis	^38^
Ten gynaecologists, six general practitioners and nine midwives aged 33–71 years (18 female and 7 male). *n* = 25	Departments of Obstetrics and Gynaecology at a university hospital, one central hospital and one private gynaecology clinic. Sweden	Women with symptoms that might indicate endometriosis	To identify and describe the experiences of healthcare professionals when meeting women with symptoms that might indicate endometriosis	^39^
Sixteen medical providers (nurse/midwife/physician), Six social workers. Two health educators (aged not specified). *n* = 24	Schools, primary care, youth clinics, residential homes for children or social services across the country. Sweden	Menstrual pain in relation to female genital cutting	To provide a qualitative exploration of how professionals in Sweden approach adolescent sexual and reproductive healthcare encounters in relation to acquired knowledge about female genital cutting, using menstrual pain as an empirical example	^56^

Notes: PCOS = polycystic ovary syndrome, HC = hormonal contraceptive, MC = menstrual cycle.

**Table 4.** Characteristics of menstruating participants from an intersectional lens

References	Age	Disability	Ethnicity/race	Gender	Migration experience	Gender identity	Sexual orientation	SES	Religion
^46^	Eight women aged 23–38 years	-	The excerpts of the women were originally documented in Swedish	Women	-	-	-	Occupations varied including a nurse, nurse’s assistant, pharmacist, office clerk, student, bus driver, and engineer.	-
^49^	Eight female junior elite players aged 16–20 years	-	-	Female athletes	-	-	-	-	-
^50^	Thirteen women aged 24–48 years	-	All of the women were native Swedish. Sweden. Inclusion criteria: speaking Swedish fluently	Women	-	-	-	Two women had completed nine years of compulsory education, four had completed upper secondary school and six women had completed university studies.	-
^55^	Twelve women aged 26–66 years	People who have been identified as having severe/profound intellectual and multiple disabilities, who require intensive support in their daily lives, and communicate with non-spoken language	-	Women	-	-	-	-	-
^51^	Nine women aged 16–22 years	-	Swedish-Somali women. Speaking Swedish or Somali using interpreter	Infibulated Swedish-Somali women	The women’s length of stay in Sweden ranged from 9 months to 6 year	-	-	Former education in Somalia or other countries before coming to Sweden ranged from a couple of months to 8 years, either in Quran schools or public schools.	
^39^	Nine women aged 23–55 years	-	Inclusion criteria were as follows: being Swedish-speaking	Women	-	-	-	One woman worked full-time, seven worked part-time (25%–80%) and one was on sick leave.	-
^40^	Sexteen female bloggers aged 22–34 years	-	-	Women	-	-	-	-	-
^48^	Thirteen elite cross-country skiers aged 20–35 years	-	Focus-group interviews were conducted in Swedish, the first language of all the participants	Female elite cross-country skiers	-	-	-	Elite cross-country skiers: University = 11. Upper secondary school = 2	-
^41^	Thirteen women aged 28–48 years	-	Twelve from Sweden and one from Europe	Women	-	-	-	University = 8. Secondary school = 4. Primary school = 1	-
^52^	Eight women aged 16–46 years	-	Women from Ethiopia, Somalia, Djibouti, and Eritrea. One of the criteria was that the participants speak enough Swedish	Women	Length of stay in Sweden was also not a criterion since we wanted to receive a wide range of experiences concerning FGM after immigration to Sweden, both from those who had lived here for some time and those who had arrived more recently			Two had finished high-school. One was studying at university to become a nurse. Educational information was not available for the others. One woman was on maternity leave.	
^53^	Twenty-three women aged 18– 65 years	-	14 originating from Somalia and 9 from Sudan. New (6): 3–12 months Single (2) Settled (17): 3–30 years	Women	The recruitment strategies selected to include informants with various lengths of stay and migration routes resulted in two groups of informants: long-term residents and newly arrived refugees	-	-	-	-
^54^	Twelve women aged 26–49 years	-	Caucasian Danish women	Women	-	All women identified themselves as women	One of the interviewees referred to herself as homosexual, seven referred to a male partner and four did not mention their sexuality or a partner	-	-
^42^	Seven women aged 21–53 years	-	Danish women	Women	-	-	-	One unemployed. One flexible job. Two students. One early retiree. One project manager. One self employed	-
^47^	Twenty-one women aged 21–36 years	-	Three of the 21 interviewees had an ethnic origin other than Danish: Iranian, Indian/Pakistani, and Kurdish	Women	-	-	One of 21 identified herself as being homosexual	Different education levels and social backgrounds.	-
^43^	Ten women aged 20–45 years	-	Women with the ability to read and write in Danish	Women	-	-	-	-	-
^44^	Twelve women aged 28–44 years	-	The inclusion criteria were being able to speak Swedish	Women	-	-	-	Their education background varied, including secondary school, further education and university education	-

Notes: Empty cells indicate characteristic was unclear/not specified/not included in this article.

### Quality assessment

No quality assessment was conducted, as this is not a requirement in scoping reviews.^37^ Furthermore, the aim of this scoping review was to map whose experiences and voices are included in or left out from menstrual health research in the Nordic countries. Thus, evaluation of the quality of the included studies was not necessary.

### Researcher characteristics and reflexivity

The research team consisted of four women who identify themselves as cis-gendered. Three are native Swedish (AW, MP MKA) and one (EÅ) was born in Thailand. EÅ has been living in the country since she was 10 years old; her father originated from Sweden and mother from Thailand. The researchers are working in a predominantly white academic environment. The entire team has research experience working with sexual and reproductive health and rights, including issues such as migration. AW is a midwife and doctoral student. MP has a background in political science and is a doctoral student. MKA is a midwife and professor in reproductive health. EÅ is a public health specialist and a postdoctoral researcher. The different professional perspectives made it possible to view the data from several angles which enriched the analysis and the interpretation of the data. Nevertheless, the researchers’ characteristics and preunderstanding and prior knowledge, as well as their own personal biases and social locations, might have influenced how they interpreted the data and discussed the findings.

## Results

### Characteristics of included studies

The search strategies used in the four databases can be found in Supplemental material. The original search in 2022 identified 2346 articles. After deduplication, the updated search identified 2733 articles (Figure 1). This means that an additional 387 articles were identified after deduplication in the updated search. A total of 22 studies met all inclusion criteria. Table 2 shows the characteristics of the included studies. The included studies spanned from 2012 to 2023, and almost half of the studies were published recently (2021–2023). The studies originated from Sweden (*n* = 13), Denmark (*n* = 5), Norway (*n* = 3), and Iceland (*n* = 1). Most publications report studies that used individual interviews (*n* = 16), mixed methods (*n* = 2), combined individual interviews and focus group discussions (*n* = 1), participant observations (*n* = 1), combined participant observation and interviews (*n* = 1), and blog posts (*n* = 1). The sample size varied between 7 and 36 people and the study population was menstruating people (*n* = 14), both menstruating people and professionals (*n* = 2), or professionals (*n* = 6). As shown in Table 2, most studies were published in a journal within the healthcare discipline.

### Types of menstrual health experiences and topics

Table 3 shows each study’s population, specific context, types of menstrual health experiences, and topics in relation to the study aim. Of all included studies, more than half reported on a topic related to menstrual disorders: eight studies focused on endometriosis^38–45^ and two on polycystic ovary syndrome (PCOS).^46,47^ Two studies focused on knowledge of the menstrual cycle and communication between athletes and their coaches.^48,49^

#### Menstruating people’s perspectives

Studies that included menstruating people included mainly people diagnosed with menstrual disorders: endometriosis (*n* = 6)^40,45^ and PCOS (*n* = 2).^46,47^ These studies focused on women’s lived experiences and circumstances related to menstrual disorders, health and quality of life, impact of treatment, and healthcare experiences. Studies including women diagnosed with endometriosis demonstrated stories that revealed a normalisation of menstrual pain by healthcare providers.^40,41^ When these women sought care for their symptoms, they were often informed that menstrual pain was normal, leading to a feeling of being mistrusted and neglected by healthcare providers. Another study explored women’s experiences of menstruation from a lifespan perspective, which included their experiences of menarche and how menstruation affected their work life and social activities.^50^ Two studies that included elite athletes’ perspectives found that they experienced difficulties communicating with their coaches about issues related to the menstrual cycle.^48,49^ Three studies focused on perceptions and experiences of female genital mutilation/cutting (FGM/C)^51–53^ and reported on menstrual pain as a negative impact of being infibulated. One study explored the connections between menstrual stigma and the use of period trackers.^54^ Björnsdóttir et al. focused on the sexuality of people with intellectual disabilities who required intensive support and communicated with non-spoken language.^54^ This study found that support staff at schools had issues with menstrual blood and one of the study participants had been required to stay home while menstruating.^55^ To solve problems regarding bleeding, this study reported that the participant received an injection with Depo-Provera without being included in the decision and consent process.

#### Professionals’ perspectives

A total of eight studies focused on professionals’ or healthcare providers’ perspectives on endometriosis (*n* = 2),^38,39^ menstrual pain (*n* = 3)^56–58^, and elite coaches’ perceptions of communication on the menstrual cycle (*n* = 2).^48,49^ Three studies did not include menstrual health in the scope of the study aim,^51,55,59^ but menstrual health experiences were reported as a secondary finding. Ahmed et al. studied care providers’ perceptions and experiences of defibulated immigrant women’s sexual and reproductive health,^59^ reporting that defibulation led to eased period experiences. Cultural norms and beliefs about menstrual pain among women that had undergone FGM/C were explored among several professionals in a study by Palm et al.^56^ The study found that most professionals described menstrual pain as a common health consequence of FGM/C. However, some professionals with clinical experience stated that there was no conclusive evidence linking menstrual pain to FGM/C and suggested that menstrual pain was common among young women irrespective of whether they had undergone FGM/C or not. In a study conducted by Höök et al.,^48^ elite coaches encountered taboos surrounding discussions about the menstrual cycle with elite athletes. They believed that such discussions were a matter of respecting the athletes’ privacy. Bergström et al.^49^ found that male elite coaches felt that they didn't know enough about menstrual cycles, so they often relied on female staff and apps to handle this communication.

#### Population characteristics and intersecting identities

Table 4 presents the study characteristics through an intersectional lens.

In all included studies, reported characteristics were used as background information on the study participants. Hence, none of the studies analysed how multiple identities intersected to shape menstrual health experiences. Intersectionality was not used as a framework in any of the included studies. The most commonly reported identity characteristics among menstruating people were age (*n* = 19), gender (*n* = 20), and SES (*n* = 15). The ages included ranged between 16 and 66 years. All studies included women and five studies targeted both women and men. Sexual orientation was reported in two studies.^47,54^ None of the included studies targeted only male participants (e.g. father, brother, male peer, or colleague) or transgender and non-binary people. Furthermore, no study included people experiencing homelessness. Three studies included participants’ ethnicity: one study targeted Somali women,^51^ the second study targeted women originating from Djibouti, Eritrea, Ethiopia, and Somalia,^52^ and the third targeted women originating from Somalia and Sudan.^53^ These studies focused mainly on views and experiences of FGM/C, and lived experiences of undergoing medical defibulation. None of the included studies reported on participants’ religion. Eight studies reported that the local language was used in interviews; some studies reported that being able to speak the local language was an inclusion criteria. Only one study included people with disability in the study population.^55^ SES was reported in fifteen studies, with a variation in included SES factors. Most studies that targeted professionals presented their occupation, whereas studies of menstruators presented their educational level.

## Discussion

This scoping review aimed to map the extent and range of qualitative research on menstrual health in the Nordic countries, using an intersectionality lens to explore whose experiences and voices were being researched. To the best of our knowledge, this is the first scoping review of qualitative research on menstrual health in the Nordic countries using an intersectional lens. We found age, gender, and SES to be the most commonly reported characteristics, and a comparative lack of consideration of people with disabilities, people with diverse gender and/or sexual identities, religions and people with a foreign background, ethnic minorities, migrants, and refugees. These characteristics were mainly reported as background variables/demographic variables or analysed independently. Thus, we have little knowledge and understanding of how characteristics intersect to shape menstrual health experiences and challenges faced by diverse groups in the Nordic countries.

Our review of study characteristics showed heterogeneity in terms of the target populations and topics of menstrual health. Studies that focused on people with experiences of the menstrual cycle had a largely medical perspective and included mainly people diagnosed with menstrual disorders such as endometriosis or PCOS. This highlights that the existing evidence base in relation to menstrual health in HICs centres mainly on menstrual disorders.^17^ The fact that the medical perspective is dominant in previous research on menstrual health leaves significant room for improvement in applying a holistic perspective to menstrual health research including an intersectional lens, so as to provide a nuanced picture. Surprisingly, none of the included studies exclusively explored the experiences of PMS although evidence shows that 20–50% of women of reproductive age experience PMS.^60^

There was an absence of studies in the Nordic countries focused on menstrual health literacy among adolescents. Given that comprehensive sex education (CSE) has strong political support and is extensively integrated in primary and secondary education in the Nordic countries,^61^ it is remarkable that research on menstrual health literacy is lacking. For example, in Sweden, CSE has been compulsory since 1955 and is integrated in the school curricula. Because CSE is part of school curricula, researching adolescents’ menstrual health literacy might seem unnecessary and this domain might be considered to be part of the school’s responsibility to address.

Further, our review reveals a lack of studies on experiences of menstrual products, which contrasts with LMIC settings where research in this area is growing.^62^ A newly published dissertation by Persdotter explored menstrual dirt and pollution in menstrual hygiene practices in Sweden, focusing on disposable pads and reusable cups. The dissertation shed light on how pollution beliefs, concealment imperatives, and stigmatisation of menstruation are enacted in everyday practices.^63^

None of the identified studies focused specifically on menstrual health among people with foreign background, migrants, refugees, and/or people belonging to ethnic minorities, despite the fact that immigration has increased in the Nordic countries in the last 10 years.^64^ The existing research on migrants’ menstrual health, although scarce, indicates that period poverty is significant. This can lead to fear and anxiety about bleeding through clothes^65^ and cause poor mental health^66^ and social effects such as harassment, isolation, and absenteeism at school.^67^ Furthermore, menstrual-related beliefs, restrictions, and gender norms can have negative impacts on women and girls’ menstrual health.^17^ Included studies in our scoping review indicated that the practice of infibulation and FGM/female genital cutting might have a negative impact on menstrual health among those exposed to it. However, two of these studies explored the perspectives of healthcare professionals and other professions (e.g. social workers and health educators), not those who had undergone FGM/C.^56,59^ The other three studies included infibulated women, who reported menstrual pain as one of many problems related to being infibulated.^51^ These findings are consistent with a previous review study that summarised empirical quantitative research describing the gynaecological consequences of FGM/C on girls and women.^68^

We found that gender and age were the most commonly reported identity characteristics in the included studies. That gender is commonly reported is unsurprising given that menstruation biologically occurs among women and girls. Similar to findings in our review, the roles of fathers, brothers, male colleagues, and peers in supporting girls and women remain under-recognised and under-researched.^25^ In our review, men’s (male coaches’) experiences and knowledge of the menstrual cycle were captured in studies within the sport context. Moreover, no studies in our review included menstruators who identified as non-binary or transgender, despite menstruation often triggering psychological concerns related to gender dysphoria.^69,70^

Furthermore, our review found a limited focus on the menstrual health of adolescents. Surprisingly, this contrasts with previous research conducted in LMICs, where major studies have focused almost exclusively on adolescent girls.^20,71^ No included studies explored adolescents’ experiences of menarche, although evidence in other HICs indicates that menarche can evoke a negative emotional response.^15,17^ Moreover, our findings demonstrate several under-researched contexts, such as the school context. The two included studies from school settings targeted professionals such as school nurses. This finding demonstrates a research gap in the Nordic countries, in contrast to the global literature where menstrual health in school contexts is a growing research area. For example, a recently published review on experiences of menstruation in schools in HICs by Thomas et al. demonstrates that menstruating students faced menstrual injustice.^72^ No studies from the Nordic countries were identified in this review. Menstruating students experienced academic disadvantage because of the failure of schools to take responsibility for menstruation management free from stigma, and to provide facilities to improve students’ capability and confidence to manage menstruation. Providing supportive facilities and services is essential for promoting menstrual health.^5^ None of our studies addressed experiences of menstrual health in workplace settings. This is surprising, as evidence from high-income countries shows that menstruation-related symptoms are associated with work absenteeism,^14,73^ yet there has been limited research on this topic in workplace settings. The growing body of research on workplace settings in HICs has focused on guidelines^74^ and menstrual leave policies.^75,76^ A review of global evidence by Howe et al. shows that the included articles report on menopause guidelines, and are less focused on how women can be supported to manage symptoms related to menstrual disorders (e.g. endometriosis and PCOS) at work.^74^ In LMICs, the scarce research has a focus on menstrual hygiene management^77–79^ and women’s menstrual experiences, and the impact of menstruation on their work.^78,80^ Menstruators spend many of their waking hours at work and school, and increased research in these settings is needed to better understand how to promote menstrual health. The World Health Organization’s (WHO) statement highlights the importance of integrating menstrual health into various parts of society, such as schools, workplaces, and healthcare systems.^81^

Furthermore, our review identified one study including people with disabilities. Previous research shows that menstruating individuals with disabilities face layers of challenges and discrimination,^22^ due to both gender norms about gender and stigma around menstruation and having a disability.^82^ Similarly to a study conducted in Nepal,^22^ our review found that people with intellectual disability who required intensive support and communicated with non-spoken language were required to stay at home while menstruating because support staff at school had issues with menstrual blood, reflecting that their rights to education are neglected.^22^ Contraceptives were provided to stop menstruation; no effort was made to include the menstruating person in the decision and consent process. This finding and those of previous studies of menstrual health among menstruating people with disabilities indicate that they have been denied their basic human rights.^83,84^ For example, one handbook published by Steele et al. highlights the case in Australia, where non-consensual sterilisation of women and girls with disabilities is legal.^84^ As Steele et al. further demonstrate “non-consensual sterilization or administering menstrual suppressant drugs would result in a violation of ICCPR Article 7” which states that no one shall be subjected to torture or cruel, inhuman, or degrading treatment or punishment. In addition to this, a systematic review by Wilbur et al. highlights that menstrual hygiene management strategies used by caregivers often involve restricting the movements of people with disabilities during menstruation and suppressing their menstruation.^85^ Of the 22 studies included in the Wilbur et al. review, 15 were from high-income countries, with one study from a Nordic setting, conducted in Denmark in 1988. The review also shows that societal beliefs and taboos surrounding menstruation and disability contribute to silence around the issue. Hennegan et al. stated in the definition of menstrual health that all menstruators should “experience a positive and respectful environment in relation to the menstrual cycle, free from stigma and psychological distress”.^5^ To ensure that menstruating people with disabilities are understood and met with dignity and respect, and free from stigma, their voices, and experiences of menstrual health need to be included in future research in the Nordic settings.

Our scoping review found an overall lack of research on menstrual health from a holistic perspective and a lack of research including people with diverse identity characteristics and social backgrounds in the Nordic countries. These findings have several implications for future research directions and policy programming and development. There is a need for a shift from a mainly medical to a holistic perspective on menstrual health, which requires interdisciplinary research and use of a broader methodological approach. A holistic approach to menstrual health is crucial as it provides a comprehensive understanding of menstruation and the menstrual cycle, addresses menstrual stigma, and promotes menstrual knowledge and inclusivity across diverse populations.^5^ The definition of menstrual health by Hennegan et al.^5^ can serve as a framework for future research and in the identification of gaps in interventions.

Furthermore, research that applies an intersectional approach is needed to enhance evidence of how different forms of social inequality affect menstrual health. Abrams et al. recommend that intersectionality be considered during study conceptualisation, as it enables researchers to account for sociohistorical forces of marginalisation and to view participant identities as multidimensional and interdependent throughout the research process.^86^ Doing this allows researchers to choose approaches, methods, and data collection and analysis strategies that are more attuned to the lived realities of participants. Increased attention is required to the needs and preferences among those whose voices are not heard, e.g. people living with disabilities, refugees, people belonging to racial and/or ethnic minority groups, people experiencing homelessness, young adolescents, men, and transgender and non-binary people. To ensure that all menstruating people are treated with dignity and respect, more evidence based on qualitative research is needed to understand their menstrual health experiences, the challenges they encounter, and the factors that promote menstrual health. Future research that addresses the experiences of menstrual health and voices among diverse groups and persons in vulnerable situations is needed as well as population-based study to quantitatively investigate menstrual health-related challenges and enabling factors.

### Strengths and limitations

One of the strengths of this scoping review is that we used broad search terms that corresponded to the holistic perspective on menstrual health,^5^ and searches were made in four databases. Furthermore, we reviewed the reference lists of the included studies to identify any potential studies that might have been missed in our search, whereby two studies were found. Nevertheless, the limitations of this study must be considered when interpreting its findings. The scoping review only included publications between 2011 and 2023. This may have meant that relevant studies published before 2011 or after 2023 were not included. Furthermore, as the literature searches were limited to peer-reviewed journals, grey literature was not included (e.g. master theses, NGO, and CBO reports). We acknowledge that this is a limitation as the review does not capture the full scope of menstrual health experience and this should be considered when interpreting the results. Another limitation is that we excluded studies related to menopause. We see this topic as relevant and with the background of using an intersectional lens, this exclusion might appear aged-biased. We would recommend future research to focus specifically on the experience of menstrual health among menstruators in their late reproductive phase. Additionally, our criteria excluded individuals with specific disorders, such as eating disorders and von Willebrand disease. This exclusion represents a limitation of our research, as it does not account for the menstrual health experiences and challenges faced by those with these conditions. Furthermore, our ability to synthesise findings through an intersectional lens might be limited, as the included studies did not conceptualise the menstrual experiences through an intersectional lens.

## Conclusions

This scoping review highlights an overall lack of research on menstrual health from a holistic perspective, and a notable gap in evidence on menstrual health experiences of menstruating people with different intersecting identities in the Nordic countries. We found that menstrual health experiences of menstruating people characterised by the following social identities were under-researched: people with disability, people of a non-Nordic ethnicity, refugees, gender-diverse people, people experiencing homelessness people and young adolescents. Thus, we have little knowledge and understanding of the experiences and challenges faced by these diverse groups in the Nordic countries, and as well the factors that promote menstrual health. Hence, more research is needed to understand experiences of menstrual health in the Nordic countries, especially research which applies an intersectional lens. The findings of this scoping review can be used to inform future research directions and policy programming.

## Supplementary Material

Table S1. Results.

Supplemental Material

Supplemental Material: Documentation of search strategies.
