# Supplementary material for: Experiences of menstrual health in the Nordic countries: a scoping review of qualitative research, applying an intersectional lens
Source: Sex Reprod Health Matters. 2025 Feb 14;32(1):2446081. doi: 10.1080/26410397.2024.2446081 (PMC11834814; doi:10.1080/26410397.2024.2446081)
Supplement: Table S1. Results. [file ZRHM_A_2446081_SM3042.docx]

| **Table S1. Extracted results** | | |
| --- | --- | --- |
| **Author and Year Published** | **Aim** | **Results** |
| Ahmed, CA. et al.  2021 | To capture care providers’ perceptions of defibulated immigrant women’s sexual and reproductive health, illuminated by their experiences as care providers for such women | Care providers perceived that woman who had been defibulated find it easier to urinate and menstruate. With a wider introitus and better passage, urination and menstruation were facilitated without pain, as well improved vaginal intercourse. It was perceived that the adaptation phase was lengthy, from being used to spending a long time urinating to having easier procedures for urination as well as menstruation. The care providers also believed that women who had undergone defibulation faced the challenge of balancing their well-being, navigating between a positive self-image and managing their emotions. Overall, the care providers perceived defibulation had a positive impact on their quality of life. |
| Angelhoff, C. et al.  2023 | To describe school nurses' experiences of supporting girls with menstrual pain | The school nurses had many experiences of supporting girls with menstrual pain. Although the school nurses took menstrual pain seriously, only a few arranged a follow-up meeting afterwards. They often recommended pain management strategies and encouraged visits to health clinics if necessary. Most nurses used to follow up the students who had severe problems, high school absenteeism and/or had been referred to other health care services. School nurses found discussing menstruation important but noted that it wasn't always comfortable for the girls. For some, the topic was sensitive and embarrassing, while others spoke about it openly. The nurses described that some girls from different cultural or ethnic backgrounds might struggle to discuss menstruation and their bodies within their families due to the topic being considered shameful and taboo. They school nurses wished for structural, national guidelines with written routines for how to treat, support, follow-up and refer girls with problems related to menstruation. |
| Bach, AM. et al.  2016 | To explore how the personal attitudes of gynaecological nurses, their specialised knowledge and their clinical experiences influenced the way they conceptualised and cared for women with endometriosis | Gynecological nurses play an important role in providing specialized care and in creating appropriate patient pathways for women undergoing major surgery for endometriosis. F  In a hospital setting, caring for women with endometriosis can pose emotional challenges for these nurses. The nurses’ attitudes toward endometriosis patients are shaped by the microculture of their workplace and their medical specialization. Factors such as biomedical knowledge, personal assumptions about endometriosis, individual values, and personal experiences with pain all impact their clinical practice. |
| Billhult, A. et al.  2012 | To describe the experience of acupuncture for women diagnosed with PCOS | The acupuncture treatment gave participants a sense of hope not previously experienced. Some participants experienced concrete results such as more regular ovulation, menstruation and even pregnancy. Participants felt a feeling of responsibility, as they discovered that the treatment had an effect on their bodies. Participants conveyed a sense of normalcy about their condition. What was once perceived as an untreatable issue with PCOS shifted to a belief in their bodies’ ability to function normally. |
| Bergström, M. et al.  2023 | To explore the perceptions of MC communication in a group of junior elite football players and their male coaches in a case study of one youth football team in a specific club in Norway | The data analysis revealed two main communication barriers: 1. interpersonal barriers (e.g., false assumptions about the coach/athletes and social discomfort) and 2. knowledge barriers (e.g., unaware/perceptions of insufficient knowledge levels). The interpersonal barriers highlight the challenges perceived by players and coaches in discussing the MC with one another, which resulted in the avoidance of MC-related communication. The second barrier, knowledge barriers, refers to how a lack of knowledge restricts both players and coaches from fully understand how the MC can affect the players' sports performance and health. |
| Brantelid, IE. et al.  2014 | To describe women’s experiences of menstruation across the lifespan | Mother plays an essential role in how participants relate to the menstrual phenomenon later in life  Menstruation is perceived as an intimate and private matter, which makes women want to conceal the occurrence of menstrual bleeding. Women find that menstruation and its symptoms reduce their concentration, patience, and efficiency, affecting their daily activities and productivity. Special bonds among women, formed through shared experiences of menstruation, create a supportive community where they can exchange knowledge and foster mutual understanding, leading to a strong sense of coherence. Over time, menstruation becomes a natural part of women’s lives and gender identity. Health professionals play a central role supporting women to deal with menstruation. |
| Björnsdóttir, K. et al.  2020 | To demonstrate how the sexuality of people with intellectual disabilities who require intensive support is shaped by sociocultural sexual scripts and the support they receive in everyday life | To prevent her menstruation from disrupting her education, she received a Depo-Provera shot every 3 months, which limited her menstrual periods. Lisa was in her twenties when we first met her, and although she was not sexually active, she still received the contraceptive shot. Support staff at her home believed the shot improved her quality of life, limited menstrual cramps and made it more manageable to assist her. No efforts were made to involve Lisa in this decision. The initial reason for the contraceptive shot was to stop Lisa’s menstruation because support staff at school had a problem with menstrual blood, which has been described as nature’s most stigmatized fluid, an abomination that is even more disgusting than seme.  […] by demanding that Lisa stayed home while menstruating, the school acknowledges that menstrual blood causes unacceptable working conditions for the support staff. |
| Chavez Karlstrom, A. et al.  2020 | To explore the lived experiences of young migrant women from Somalia and their views on undergoing medical defibulation in Sweden | […] stating problems they themselves related to their infibulation as menstrual pains, but at the same time wished to follow the tradition and maintain their own infibulated state in order to prove their virginity at the time of marriage.  […] she had severe pains while menstruating, but firmly stated that while she sometimes overheard her mother discuss with friends the importance of stopping FGC and infibulation, they never discussed the issue of medical defibulation at home. |
| Eldestrand, L. et al.  2022 | To describe midwives’ experiences of supporting girls with menstrual pain | The midwives felt confident in their role of helping to relieve the menstrual pain of young women. Hormonal contraceptives were often a natural choice and an effective method for pain relief, although the midwives occasionally faced resistance from young women or their mothers when recommending this. They consulted or referred to a gynecologist if pain relief was not achieved. The midwives’ experiences highlight that young women often have gaps in their understanding of menstrual pain and its relief methods. They emphasized the need to improve young women’s knowledge about the menstrual cycle and the physiology of menstrual pain to help them better grasp its causes and management. |
| Grundstrom, H. et al.  2015 | To identify and describe the experiences of healthcare professionals when meeting women with symptoms that might indicate endometriosis | Healthcare professionals tried to create a supportive encounter by recognizing the woman's concerns, listening attentively, and explaining the issues. The healthcare professionals found women with symptoms that could indicate endometriosis challenging to address, as they required a high amount of responsiveness. They had varied approaches to defining what was normal regarding menstrual pain, ovulation pain, and dyspareunia. They also needed to have the competence to act and react when the symptoms indicated endometriosis. There was consensus among the GPs and midwives that the gynecologist was the one who should inform and treat women with presumed endometriosis. |
| Grundström, H. et al.  2017 | To identify and describe the experience of healthcare encounters among women with endometriosis | All women had encountered both destructive or constructive experiences, though some had more of one type than the other. The destructive side was characterised by ignorance, exposure and disbelief. The constructive side made the women feel acknowledged and confirmed, boosting their self-esteem. Women experienced mental and physical exposure, diagnostic delay, and the normalization of symptoms as part of normal life.  Since the disease impacted their entire lives, their healthcare interactions were crucial, as the responses they received from healthcare encounters shaped their thoughts and perceptions about their bodies. |
| Grundström, H. et al.  2020 | To identify and describe endometriosis healthcare experiences based on affected individuals’ blog posts. | The bloggers described their journey through endometriosis healthcare as a protracted struggle,  that continually misinterpreted, normalised and trivialised endometriosis symptoms. In order to get proper help, they had to seek care repeatedly. The bloggers felt dependent on the healthcare professionals’ response they received when disclosing their symptoms. In the bloggers’ experience, healthcare professionals’ knowledge about endometriosis varied. Positive encounters were often characterised by continuity, a person-centred approach and the healthcare professionals’ high degree of competence. Lack of competence led to what the bloggers considered incorrect treatments, and not having their care needs fulfilled. |
| Hook, M. et al.  2021 | To gain an in-depth understanding of the perceptions and experiences of elite female endurance athletes and their coaches in relation to barriers to communication about MC and HC issues | Three main barriers to communication were identified: knowledge, interpersonal, and structural. Perceived low levels of knowledge hindered communication between athletes and coaches. A strong coach–athlete relationship seems to be one factor that may reduce the barriers to communication. Athletes did not feel that hormonal cycles affected their health or sporting performance negatively, and therefore felt no need to discuss the subject with their coaches. Although most athletes had questions and experiences related to female hormonal cycles and elite sport, they seldom discussed these issues with their teammates. Many of the athletes in the believed that MC- and HC-related issues were outside the knowledge area of their coaches. Although all the coaches talked about MC or HC with their athletes to some extent, they, especially the male coaches, often felt insecure and uncomfortable during these conversations. |
| Hållstam, A. et al.  2018 | To explore how persons with endometriosis experienced their health after dietary changes | The endometriosis influenced the women’s sensations, feelings and reactions, creating a sense of difference (Woman with painful endometriosis). The condition induced dependence on health care and significant others and these encounters could be helpful or harmful (Dependence). Further overall physical, social and existential consequences were described (A ruined life). Endometriosis led to lost opportunities in education and career, forcing some women to work part-time or even lose their jobs. The economic impact was worsened by the costs of treatment, resulting in feeling oc double punishment. To cope with this over whelming situation the women had to struggle for coherence by searching for understanding, coping and meaning, shown in the core category (Living with painful endometriosis). |
| Isman, E., et al. 2013 | To explore how women from part of the world where female genital mutilation (FGM) is normative perceive and experience FGM after immigrating to Sweden | All the women who had been infibulated described the procedure as having negatively affected their lives. They had experienced painful menstruations and dyspareunia. |
| Karlsson, A.  2019 | To explore menstrual stigma and the usage of period trackers and investigate how digital traces from datafied bodies transmit meaning in everyday life | The women in the study described how the app provides them with reassurance and privacy. The period apps are experienced as private, shame-free rooms for exploratory engagement with the menstruating body. The participants experienced the period tracker as a way of reclaiming the body – using algorithmic predictions to make sense of their everyday life, be it by individual engagement with the app, by sending screen dumps of PMS notifications to a partner or by comparing unstable cycles with peers. The risk of embodied data potentially becoming shareable commodities does not affect the everyday self-tracking practice of these women. |
| Nielsen, LJ. et al.  2022 | To explore the lived experiences of endometriosis in adolescence and the social reactions impact on the illness experience and quality of life | The women's narratives are characterised into a before and after diagnosis. The women felt that doctors in the healthcare system failed to provide the necessary help and treatment for their severe pain, leading them to feel neglected and dismissed in their attempts to have their illness taken seriously. Being rejected by health professionals further resulted in the informants hiding their symptoms from others. Most informants reported that the lack of recognition from both school and friends was central to their experience. They described how the normalization of menstruation-related issues by their school and social circles contributed to a failure to acknowledge endometriosis. Despite different ways of coping with the illness, all informants had a shared narrative about coping that included ways of controlling fears and the extent to which endometriosis affected their quality of life. |
| Pfister, G. et al.  2017 | “It's not very feminine to have a mustache”: Experiences of Danish women with polycystic ovary syndrome | The women with PCOS often felt that their bodies deviated from societal norms of femininity, using terms such as “different,” “wrong,” “unnatural,” “abnormal,” “unfeminine,” and “masculine” to describe their experiences. They perceived their bodies as different because of the symptoms of PCOS, namely, hirsutism. Hirsutism had a decisive negative influence on the women’s everyday lives, particularly with regard to male partners and sexual relations. Several of the women reported about negative experiences with health professionals, in particular, when they consulted them about hirsutism. Some doctors seemed to have a lack of expertise about PCOS or were unwilling to take their symptoms seriously.  They used different strategies, such as hair removal and covering the body, to live up to body ideals and cope with the symptoms. |
| Palm, G. et al., 2022 | To provide a qualitative exploration of how professionals in Sweden approach adolescent sexual and reproductive healthcare encounters in relation to acquired knowledge about female genital cutting, using menstrual pain as an empirical example | Most professionals presented menstrual pain as a common health consequence of FGC. The Somali group was the group repeatedly referred to in the professionals’ accounts. Some stated that menstrual pain was a ‘very common complaint’ in all young women they had encountered, that they ‘wouldn’t be able to tell the difference between young women with or without FGC. There was a tendency in counselling to differentiate young migrant women’s menstrual complaints from ordinary menstrual pain, with professionals understanding pain complaints either in terms of FGC or as culturally influenced. Counselling was also shaped by biomedical knowledge as well as culture-specific expectations and assumptions about menstrual pain. They sympathised with the young women’s strategies during menstruation, acknowledging that all young women might experience stigma and challenges in continuing life as usual during menstruation. |
| Johansen, R.E., 2017 | To explores experiences and perceptions of premarital defibulation | The relief of being able to urinate and menstruate normally was only one aspect of have been difibulated. Equally important, according to one participant, was a sense of regaining control over her body. |
| Simonsen, SM. et al.  2023 | To assess changes in self-reported endometriosis-specific health-related quality of life (HRQOL) and confidence in managing health and care | Participants felt alone with the disease. They described trying different methods to receive help with their condition but had not succeeded in getting what they needed. The participants had several worries regarding fertility, sexuality, their relationship to their partner and having to take sick leave due to pain. According to the participants, the intervention fostered meaningful relationships with healthcare professionals and provided person-specific knowledge that facilitated new behaviours. They also began to accept living with endometriosis and its persistent symptoms. |
| Vennberg Karlsson, J. et al.  2020 | To explore how persons with endometriosis experienced their health after dietary changes | Participants experienced an increase in wellbeing and a decrease in symptoms after the dietary changes. They also experienced increased energy and gained a better understanding of how their dietary changes impacted their health by paying attention to their body’s responses. The dietary change posed several challenges. Meal planning was time-consuming, including the difficulty of maintaining. Meal planning was time-consuming and constantly having to think about what to eat was exhausting. Another challenge in maintaining dietary changes was the lengthy period, sometimes weeks or even years, before experiencing positive effects. Support from family and friends was important in implementing and sustaining the dietary changes. However, the participants stressed the lack of support from healthcare professionals. |
