# Supplementary material for: Experiences of menstrual health in the Nordic countries: a scoping review of qualitative research, applying an intersectional lens
Source: Sex Reprod Health Matters. 2025 Feb 14;32(1):2446081. doi: 10.1080/26410397.2024.2446081 (PMC11834814; doi:10.1080/26410397.2024.2446081)
Supplement: Supplemental Material [file ZRHM_A_2446081_SM3040.docx]

| **Table S2. Characteristics of professionals from an intersectional lens** | | | | | | | | | |
| --- | --- | --- | --- | --- | --- | --- | --- | --- | --- |
| **References** | **Age** | **Disability** | **Ethnicity/race** | **Gender** | **Gender identity** | **Migration experience** | **Sexual orientation** | **Socio-economic status** | **Religion** |
| Ahmed, CA. et al.  2021 | Seven midwives and six gynaecologists aged 45-65 years | - | The interviews were conducted in Swedish | Women and men | - | - | - | Midwives gynaecologists | - |
| Angelhoff, C. et al.  2023 | Fifteen nurses aged 36–58 years | - | - | Women and men | - | - | - | Nurses  Midwives | - |
| Bach, AM. et al.  2016 | Nine gynaecological nurses aged 24–61 years | - | - | Women | - | - | - | Bachelor’s degree in nursing, several had further education | - |
| Bergström, M. et al.  2023 | Two coaches  (aged not specified) | - | - | Men | - | - | - | - | - |
| Eldestrand, L. et al.  2022 | Fifteen midwives aged 28–63 years | - | - | Women | - | - | - | Midwives | - |
| Grundstrom, H. et al.  2015 | Twenty-five healthcare professionals aged 31–71 years | - | HCP from Sweden | Women  Men | - | - | - | Gynaecologists, GPs,  midwives | - |
| Hook, M. et al.  2021 | Thirteen elite cross-country coaches aged 30-60 years | - | -  Focus-group interviews were conducted in Swedish, the first language of all the participants | Female elite cross-country skiers and eight of their coaches (two women and six men) | - | - | - | Coaches: University = 8 | - |
| Palm, C. et al 2022 | Sixteen medical providers (aged not specified) | - | - | Women | - | - | - | 16 medical providers  6 social workers  2 health educators | - |
