# Supplementary material for: Experiences of menstrual health in the Nordic countries: a scoping review of qualitative research, applying an intersectional lens
Source: Sex Reprod Health Matters. 2025 Feb 14;32(1):2446081. doi: 10.1080/26410397.2024.2446081 (PMC11834814; doi:10.1080/26410397.2024.2446081)
Supplement: Supplemental Material: Documentation of search strategies. [file ZRHM_A_2446081_SM8431.docx]

Documentation of search strategies

University Library search consultation group

Date: January 2022, updated June 2023

Topic/research question: Menstrual health in the Nordic countries

Name of researcher(s): Eva Åkerman and Anna Wängborg, Institutionen för kvinnors och barns hälsa

Librarian(s): Sabina Gillsund & Jonas Pettersson

Databases:

1. Medline (Ovid)
2. Web of Science (Clarivate Analytics)
3. PsycInfo (EBSCO)
4. CINAHL (EBSCO)

Total number of hits:

- Before deduplication: 5,207
- After deduplication: 2733

Text that can be used in the Methods-section:

Search strategy

A literature search was performed in the following databases: Medline, Web of Science, PsycInfo and CINAHL.

After the original search was performed on 26 January 2022, the search was last updated on 9 June 2023 using the methods described by Bramer et al (1).

The final search strategy was developed in collaboration with an information specialist at the Karolinska Institutet University Library. The initial search strategy was developed in Medline, where MeSH terms and free-text terms were identified for each search concept. These terms were then translated into the other databases using Polyglot Search Translator (2). The search strategy was subsequently adapted for databases Web of Science, PsycInfo, and CINAHL. This process involved not

only translating the syntax but also converting MeSH terms into their equivalents, such as CINAHL Headings and APA Thesaurus of Psychological Index Terms.

The strategies were peer reviewed by another librarian prior to execution.

De-duplication was done using the method described by Bramer et al (3). One final, extra step was added to compare DOIs.

1. Medline

| Interface: Ovid MEDLINE(R) ALL  Date of Search: June 9, 2023  Number of hits: 1851  Comment: In Ovid, two or more words are automatically searched as phrases; i.e. no quotation marks are needed  The Ovid MEDLINE®️ database contains records with the following possible status besides MEDLINE: Publisher, In-Data-Review, In-Process and PubMed-not-MEDLINE records from NLM. | Field labels   - exp/ = exploded MeSH term - / = non exploded MeSH term - .ti,ab,kf. = title, abstract and author keywords - adjx = within x words, regardless of order - * = truncation of word for alternate endings |
| --- | --- |
| Database(s): **Ovid MEDLINE(R) ALL**1946 to June 08, 2023 Search Strategy:   \| **#** \| **Searches** \| **Results** \| \| --- \| --- \| --- \| \| 1 \| Menarche/ \| 5418 \| \| 2 \| exp Menstrual Cycle/ \| 35564 \| \| 3 \| exp Menstruation Disturbances/ \| 29293 \| \| 4 \| Menstrual Hygiene Products/ \| 631 \| \| 5 \| Endometriosis/ \| 25416 \| \| 6 \| Polycystic Ovary Syndrome/ \| 17829 \| \| 7 \| (abnormal uterine bleeding or amenorrhea* or dysmenorrhea* or endometrioma* or endometriosis or heavy period* or hypomenorrhea or irregular uterine bleeding or menarche or menorrhagia or menstru* or menses or oligomenorrhea* or polymenorrhea or polycystic ovary syndrome or premenstrual or sclerocystic ovar* or stein leventhal syndrome or tampon?).ti,ab,kf. \| 127194 \| \| 8 \| 1 or 2 or 3 or 5 or 6 or 7 \| 155095 \| \| 9 \| "Quality of Life"/ \| 266808 \| \| 10 \| Life Style/ \| 63536 \| \| 11 \| exp Adaptation, Psychological/ \| 139483 \| \| 12 \| Mental Health/ \| 60844 \| \| 13 \| Sexual Health/ \| 2426 \| \| 14 \| Women's Health/ \| 29521 \| \| 15 \| Anxiety/ \| 106105 \| \| 16 \| Health Behavior/ \| 56367 \| \| 17 \| Social Stigma/ \| 12726 \| \| 18 \| exp Shame/ \| 3010 \| \| 19 \| Attitude/ \| 53579 \| \| 20 \| "Attitude to Health"/ \| 85420 \| \| 21 \| Health Knowledge, Attitudes, Practice/ \| 126419 \| \| 22 \| Depression/ \| 150031 \| \| 23 \| exp Emotions/ \| 406926 \| \| 24 \| exp Stress, Psychological/ \| 152451 \| \| 25 \| Pain/ \| 152368 \| \| 26 \| Pain Measurement/ \| 94503 \| \| 27 \| Pain Perception/ \| 3181 \| \| 28 \| exp Exercise/ \| 245297 \| \| 29 \| Health Status/ \| 89864 \| \| 30 \| Psychosocial Functioning/ \| 371 \| \| 31 \| exp Work/ \| 70193 \| \| 32 \| exp Education/ \| 898328 \| \| 33 \| exp Feeding Behavior/ \| 192338 \| \| 34 \| exp Diet/ \| 327664 \| \| 35 \| exp Sleep/ \| 97293 \| \| 36 \| exp Sleep Wake Disorders/ \| 108577 \| \| 37 \| therapy.fs. \| 2119581 \| \| 38 \| drug therapy.fs. \| 2596988 \| \| 39 \| (anxi* or attitude* or behavior* or behaviour* or cope or coping or depress* or diet* or eating or emotion* or education* or exercise or experience* or feeding or food? or information or knowledge or lifestyle or life style or manage* or "quality of life" or pain or perception* or practice* or psychological adaptation or psychosocial or psycho-social or physical activit* or shame* or sleep* or stigma* or strateg* or stress or therap* or treatment* or view* or wellbeing or well-being or work*).ti,ab,kf. \| 15893130 \| \| 40 \| ((female* or mental* or menstru* or psycho* or reproductive or sexual* or status or woman or women*) adj3 health*).ti,ab,kf. \| 464562 \| \| 41 \| or/9-40 \| 17808260 \| \| 42 \| exp "Scandinavian and Nordic Countries"/ \| 220525 \| \| 43 \| (denmark or danish or finland or finnish or greenland* or iceland* or faroe islands or faeroe islands or nordic countr* or norway or norwegian or scandinavia* or sweden or swedish).ti,ab,kf,in. \| 1016847 \| \| 44 \| 42 or 43 \| 1072227 \| \| 45 \| 8 and 41 and 44 \| 3966 \| \| 46 \| limit 45 to yr="2011 -Current" \| 1851 \| | |

2. Web of Science Core Collection

| Interface: Clarivate Analytics  Editions = A&HCI , ESCI , SCI-EXPANDED , SSCI  Date of Search: June 9, 2023  Number of hits: 2,286 | Field labels   - TS/Topic = title, abstract, author keywords and Keywords Plus - NEAR/x = within x words, regardless of order - * = truncation of word for alternate endings   Note: the *Exact search*-function was used for all the searches |
| --- | --- |
| \| **No** \| **Search Query** \| **Results** \| \| --- \| --- \| --- \| \| #1 \| TS=("abnormal uterine bleeding" OR amenorrhea* OR dysmenorrhea* OR endometrioma* OR endometriosis OR "heavy period*" OR hypomenorrhea OR "irregular uterine bleeding" OR menarche OR menorrhagia OR menstru* OR menses OR menstrual OR oligomenorrhea* OR polymenorrhea OR "polycystic ovary syndrome" OR premenstrual OR "sclerocystic ovar*" OR "stein leventhal syndrome" OR tampon$ ) \| 126337 \| \| #2 \| TS=((female* OR mental* OR menstru* OR psycho* OR reproductive OR sexual* OR status OR woman OR women* ) NEAR/3 health* ) \| 589158 \| \| #3 \| TS=(anxi* OR attitude* OR behavior* OR behaviour* OR cope OR coping OR depress* OR diet* OR eating OR emotion* OR education* OR exercise OR experience* OR feeding OR food$ OR information OR knowledge OR lifestyle OR "life style" OR manage* OR "quality of life" OR pain OR perception* OR practice* OR "psychological adaptation" OR psychosocial OR "psycho-social" OR "physical activit*" OR shame* OR sleep* OR stigma* OR strateg* OR stress OR therap* OR treatment* OR view* OR wellbeing OR well-being OR work* ) \| 26088309 \| \| #4 \| #2 OR #3 \| 26186683 \| \| #5 \| AD=(denmark OR danish OR finland OR finnish OR greenland* OR iceland* OR "faraoe islands" OR "nordic countr*" OR norway OR norwegian OR scandinavia* OR sweden OR swedish) \| 2350798 \| \| #6 \| TS=(denmark OR danish OR finland OR finnish OR greenland* OR iceland* OR "faraoe islands" OR "nordic countr*" OR norway OR norwegian OR scandinavia* OR sweden OR swedish) \| 465557 \| \| #7 \| #5 OR #6 \| 2499872 \| \| #8 \| #1 AND #4 AND #7 and 2023 or 2022 or 2021 or 2020 or 2019 or 2018 or 2017 or 2016 or 2015 or 2014 or 2013 or 2012 or 2011 (Publication Years) \| 2286 \| | |

3. Psycinfo

| Interface: EBSCO  Date of Search: June 9, 2023  Number of hits: 213 | Field labels   - DE = subject heading - TI = title - AB = abstract - KW = author keywords - Nx = within x words, regardless of order - * = truncation of word for alternate endings |
| --- | --- |
| \| **#** \| **Query** \| **Results** \| \| --- \| --- \| --- \| \| S33 \| S4 AND S30 AND S31 Limiters - Publication Year: 2011-2023 \| 213 \| \| S32 \| S4 AND S30 AND S31 \| 498 \| \| S31 \| (denmark OR danish OR finland OR finnish OR greenland* OR iceland* OR "faroe islands" OR "faeroe islands" OR "nordic countr*" OR norway OR norwegian OR scandinavia* OR sweden OR swedish) \| 190,941 \| \| S30 \| S5 OR S6 OR S7 OR S8 OR S9 OR S10 OR S11 OR S12 OR S13 OR S14 OR S15 OR S16 OR S17 OR S18 OR S19 OR S20 OR S21 OR S22 OR S23 OR S24 OR S25 OR S26 OR S27 OR S28 OR S29 \| 4,291,795 \| \| S29 \| TI ( ((female* OR mental* OR menstru* OR psycho* OR reproductive OR sexual* OR status OR woman OR women*) N3 health*) ) OR AB ( ((female* OR mental* OR menstru* OR psycho* OR reproductive OR sexual* OR status OR woman OR women*) N3 health*) ) OR KW ( ((female* OR mental* OR menstru* OR psycho* OR reproductive OR sexual* OR status OR woman OR women*) N3 health*) ) \| 327,856 \| \| S28 \| TI ( (anxi* OR attitude* OR behavior* OR behaviour* OR cope OR coping OR depress* OR diet* OR eating OR emotion* OR education* OR exercise OR experience* OR feeding OR food* OR information OR knowledge OR lifestyle OR "life style" OR manage* OR "quality of life" OR pain OR perception* OR practice* OR "psychological adaptation" OR psychosocial OR psycho-social OR "physical activit*" OR shame* OR sleep* OR stigma* OR strateg* OR stress OR therap* OR treatment* OR view* OR wellbeing OR well-being OR work*) ) OR AB ( (anxi* OR attitude* OR behavior* OR behaviour* OR cope OR coping OR depress* OR diet* OR eating OR emotion* OR education* OR exercise OR experience* OR feeding OR food* OR information OR knowledge OR lifestyle OR "life style" OR manage* OR "quality of life" OR pain OR perception* OR practice* OR "psychological adaptation" OR psychosocial OR psycho-social OR "physical activit*" OR shame* OR sleep* OR stigma* OR strateg* OR stress OR therap* OR treatment* OR view* OR wellbeing OR well-being OR work*) ) OR KW ( (anxi* OR attitude* OR behavior* OR behaviour* OR cope OR coping OR depress* OR diet* OR eating OR emotion* OR education* OR exercise OR experience* OR feeding OR food* OR information OR knowledge OR lifestyle OR "life style" OR manage* OR "quality of life" OR pain OR perception* OR practice* OR "psychological adaptation" OR psychosocial OR psycho-social OR "physical activit*" OR shame* OR sleep* OR stigma* OR strateg* OR stress OR therap* OR treatment* OR view* OR wellbeing OR well-being OR work*) ) \| 4,214,360 \| \| S27 \| DE "Life Experiences" OR DE "Life Changes" \| 33,880 \| \| S26 \| DE "Sleep Wake Disorders" OR DE "Hypersomnia" OR DE "Insomnia" OR DE "Narcolepsy" OR DE "Parasomnias" OR DE "Sleep Apnea" OR DE "Bruxism" OR DE "REM Sleep Behavior Disorder" OR DE "Restless Leg Syndrome" OR DE "Sleep Arousal Disorders" OR DE "Sleep Terrors" OR DE "Sleepwalking" OR DE "Sleep-Related Hypoventilation" \| 28,257 \| \| S25 \| (DE "Sleep" OR DE "Dreaming" OR DE "Napping" OR DE "NREM Sleep" OR DE "REM Sleep" OR DE "Sleep Onset" OR DE "Sleep Quality" OR DE "Snoring" OR DE "Dream Content" OR DE "Dream Recall" OR DE "Lucid Dreaming" OR DE "Nightmares" OR DE "REM Dreams" OR DE "REM Dream Deprivation") \| 48,424 \| \| S24 \| DE "Diets" OR DE "Meat Consumption" OR DE "Vegan Diet" OR DE "Vegetarian Diet" OR DE "Weight Control" \| 20,160 \| \| S23 \| DE "Eating Behavior" OR DE "Binge Eating" OR DE "Bottle Feeding" OR DE "Breast Feeding" OR DE "Chewing" OR DE "Dietary Restraint" OR DE "Emotional Eating" OR DE "Food Refusal" OR DE "Healthy Eating" OR DE "Weaning" \| 27,651 \| \| S22 \| DE "Life Satisfaction" OR DE "Work-Life Balance" \| 13,942 \| \| S21 \| DE "Psychosocial Factors" \| 37,763 \| \| S20 \| DE "Health Status" \| 22,524 \| \| S19 \| DE "Exercise" OR DE "Aerobic Exercise" OR DE "Weightlifting" OR DE "Yoga" \| 40,391 \| \| S18 \| DE "Pain Perception" \| 11,873 \| \| S17 \| DE "Pain Measurement" \| 16,813 \| \| S16 \| DE "Pain" \| 43,778 \| \| S15 \| DE "Perceived Stress" \| 2,197 \| \| S14 \| DE "Health Attitudes" \| 11,373 \| \| S13 \| DE "Attitudes" \| 31,118 \| \| S12 \| DE "Stigma" \| 17,150 \| \| S11 \| DE "Health Behavior" \| 43,094 \| \| S10 \| (DE "Emotional States" OR DE "Affection" OR DE "Agitation" OR DE "Alienation" OR DE "Ambivalence" OR DE "Apathy" OR DE "Belonging" OR DE "Boredom" OR DE "Catastrophizing" OR DE "Compassion" OR DE "Contentment" OR DE "Depression (Emotion)" OR DE "Disappointment" OR DE "Disgust" OR DE "Dissatisfaction" OR DE "Distress" OR DE "Doubt" OR DE "Embarrassment" OR DE "Emotional Exhaustion" OR DE "Emotional Trauma" OR DE "Empathy" OR DE "Enthusiasm" OR DE "Euphoria" OR DE "Euthymia" OR DE "Frustration" OR DE "Gratitude" OR DE "Greed" OR DE "Grief" OR DE "Guilt" OR DE "Happiness" OR DE "Homesickness" OR DE "Hope" OR DE "Hopelessness" OR DE "Jealousy" OR DE "Loneliness" OR DE "Love" OR DE "Mental Confusion" OR DE "Optimism" OR DE "Passion" OR DE "Pessimism" OR DE "Pleasure" OR DE "Psychological Capital" OR DE "Regret" OR DE "Restlessness" OR DE "Sadness" OR DE "Shame" OR DE "Solidarity" OR DE "Suffering" OR DE "Suspicion" OR DE "Sympathy" OR DE "Anger" OR DE "Anger Expression" OR DE "Hostility" OR DE "Anxiety" OR DE "Anxiety Sensitivity" OR DE "Climate Anxiety" OR DE "Computer Anxiety" OR DE "Death Anxiety" OR DE "Health Anxiety" OR DE "Mathematics Anxiety" OR DE "Performance Anxiety" OR DE "Social Anxiety" OR DE "Speech Anxiety" OR DE "Test Anxiety" OR DE "Travel Anxiety" OR DE "Aversion" OR DE "Hate" OR DE "Bereavement" OR DE "Grief" OR DE "Fear" OR DE "Fear of Success" OR DE "Panic" OR DE "Helplessness" OR DE "Learned Helplessness" OR DE "Mania" OR DE "Hypomania" OR DE "Morale" OR DE "Demoralization" OR DE "Pride" OR DE "Patriotism" OR DE "Psychological Engagement" OR DE "Employee Engagement" OR DE "Student Engagement") \| 397,975 \| \| S9 \| DE "Sexual Health" \| 3,423 \| \| S8 \| DE "Mental Health" OR DE "Athlete Mental Health" OR DE "Youth Mental Health" \| 97,095 \| \| S7 \| DE "Coping Behavior" OR DE "Coping Style" \| 54,298 \| \| S6 \| DE "Quality of Life" OR DE "Health Related Quality of Life" OR DE "Quality of Work Life" \| 69,671 \| \| S5 \| DE "Lifestyle" \| 12,190 \| \| S4 \| S1 OR S2 OR S3 \| 12,568 \| \| S3 \| TI ( ("abnormal uterine bleeding" OR amenorrhea* OR dysmenorrhea* OR endometrioma* OR endometriosis OR "heavy period*" OR hypomenorrhea OR "irregular uterine bleeding" OR menarche OR menorrhagia OR menstru* OR menses OR oligomenorrhea* OR polymenorrhea OR "polycystic ovary syndrome" OR premenstrual OR "sclerocystic ovar*" OR "stein leventhal syndrome" OR tampon*) ) OR AB ( ("abnormal uterine bleeding" OR amenorrhea* OR dysmenorrhea* OR endometrioma* OR endometriosis OR "heavy period*" OR hypomenorrhea OR "irregular uterine bleeding" OR menarche OR menorrhagia OR menstru* OR menses OR oligomenorrhea* OR polymenorrhea OR "polycystic ovary syndrome" OR premenstrual OR "sclerocystic ovar*" OR "stein leventhal syndrome" OR tampon*) ) OR KW ( ("abnormal uterine bleeding" OR amenorrhea* OR dysmenorrhea* OR endometrioma* OR endometriosis OR "heavy period*" OR hypomenorrhea OR "irregular uterine bleeding" OR menarche OR menorrhagia OR menstru* OR menses OR oligomenorrhea* OR polymenorrhea OR "polycystic ovary syndrome" OR premenstrual OR "sclerocystic ovar*" OR "stein leventhal syndrome" OR tampon*) ) \| 11,514 \| \| S2 \| DE "Menstrual Disorders" OR DE "Amenorrhea" OR DE "Dysmenorrhea" OR DE "Premenstrual Dysphoric Disorder" \| 1,639 \| \| S1 \| (DE "Menstrual Cycle" OR DE "Menstruation" OR DE "Ovulation" OR DE "Menarche") \| 5,609 \| | |

4. Cinahl

| Interface: Ebsco  Date of Search: June 9, 2023  Number of hits: 857 | Field labels   - MH+ = exploded Cinahl Heading - MH = non exploded Cinahl Heading - TI = title - AB = abstract - Nx = within x words, regardless of order - * = truncation of word for alternate endings |
| --- | --- |
| \| **#** \| **Query** \| **Results** \| \| --- \| --- \| --- \| \| S46 \| S8 AND S42 AND S45 \| 857 \| \| S45 \| S43 OR S44 \| 245,064 \| \| S44 \| TI ( (denmark OR danish OR finland OR finnish OR greenland* OR iceland* OR "faroe islands" OR "faeroe islands" OR "nordic countr*" OR norway OR norwegian OR scandinavia* OR sweden OR swedish) ) OR AB ( (denmark OR danish OR finland OR finnish OR greenland* OR iceland* OR "faroe islands" OR "faeroe islands" OR "nordic countr*" OR norway OR norwegian OR scandinavia* OR sweden OR swedish) ) OR AF ( (denmark OR danish OR finland OR finnish OR greenland* OR iceland* OR "faroe islands" OR "faeroe islands" OR "nordic countr*" OR norway OR norwegian OR scandinavia* OR sweden OR swedish) ) \| 236,475 \| \| S43 \| (MH "Iceland") OR (MH "Scandinavia+") \| 82,580 \| \| S42 \| S9 OR S10 OR S11 OR S12 OR S13 OR S14 OR S15 OR S16 OR S17 OR S18 OR S19 OR S20 OR S21 OR S22 OR S23 OR S24 OR S25 OR S26 OR S27 OR S28 OR S29 OR S30 OR S31 OR S32 OR S33 OR S34 OR S35 OR S36 OR S37 OR S38 OR S39 OR S40 OR S41 \| 4,854,351 \| \| S41 \| TI ( ((female* OR mental* OR menstru* OR psycho* OR reproductive OR sexual* OR status OR woman OR women*) N3 health*) ) OR AB ( ((female* OR mental* OR menstru* OR psycho* OR reproductive OR sexual* OR status OR woman OR women*) N3 health*) ) \| 261,704 \| \| S40 \| TI ( (anxi* OR attitude* OR behavior* OR behaviour* OR cope OR coping OR depress* OR diet* OR eating OR emotion* OR education* OR exercise OR experience* OR feeding OR food* OR information OR knowledge OR lifestyle OR "life style" OR manage* OR "quality of life" OR pain OR perception* OR practice* OR "psychological adaptation" OR psychosocial OR psycho-social OR "physical activit*" OR shame* OR sleep* OR stigma* OR strateg* OR stress OR therap* OR treatment* OR view* OR wellbeing OR well-being OR work*) ) OR AB ( (anxi* OR attitude* OR behavior* OR behaviour* OR cope OR coping OR depress* OR diet* OR eating OR emotion* OR education* OR exercise OR experience* OR feeding OR food* OR information OR knowledge OR lifestyle OR "life style" OR manage* OR "quality of life" OR pain OR perception* OR practice* OR "psychological adaptation" OR psychosocial OR psycho-social OR "physical activit*" OR shame* OR sleep* OR stigma* OR strateg* OR stress OR therap* OR treatment* OR view* OR wellbeing OR well-being OR work*) ) \| 4,053,715 \| \| S39 \| MW "TH" \| 653,705 \| \| S38 \| (MH "Sleep Disorders+") \| 45,069 \| \| S37 \| (MH "Sleep+") \| 32,254 \| \| S36 \| (MH "Diet+") \| 137,625 \| \| S35 \| (MH "Eating Behavior+") \| 49,552 \| \| S34 \| (MH "Education+") \| 998,912 \| \| S33 \| (MH "Work-Life Balance") \| 3,031 \| \| S32 \| (MH "Work+") \| 9,092 \| \| S31 \| (MH "Psychosocial Functioning") \| 332 \| \| S30 \| (MH "Health Status+") \| 131,547 \| \| S29 \| (MH "Exercise+") \| 127,432 \| \| S28 \| (MH "Pain Measurement") \| 52,230 \| \| S27 \| (MH "Pain") \| 79,565 \| \| S26 \| (MH "Stress, Psychological+") \| 100,998 \| \| S25 \| (MH "Emotions+") \| 174,168 \| \| S24 \| (MH "Depression") \| 127,314 \| \| S23 \| (MH "Health Knowledge") \| 36,455 \| \| S22 \| (MH "Attitude to Health") \| 48,800 \| \| S21 \| (MH "Attitude") \| 17,464 \| \| S20 \| (MH "Shame+") \| 2,900 \| \| S19 \| (MH "Stigma") \| 20,823 \| \| S18 \| (MH "Health Behavior") \| 57,802 \| \| S17 \| (MH "Anxiety") \| 57,291 \| \| S16 \| (MH "Women's Health") \| 46,358 \| \| S15 \| (MH "Sexual Health") \| 8,524 \| \| S14 \| (MH "Mental Health") \| 54,487 \| \| S13 \| (MH "Coping+") \| 42,511 \| \| S12 \| (MH "Adaptation, Psychological+") \| 39,255 \| \| S11 \| (MH "Life Experiences+") \| 56,298 \| \| S10 \| (MH "Life Style") \| 29,681 \| \| S9 \| (MH "Quality of Life+") \| 148,029 \| \| S8 \| S1 OR S2 OR S3 OR S4 OR S5 OR S6 OR S7 \| 40,775 \| \| S7 \| TI ( ("abnormal uterine bleeding" OR amenorrhea* OR dysmenorrhea* OR endometrioma* OR endometriosis OR "heavy period*" OR hypomenorrhea OR "irregular uterine bleeding" OR menarche OR menorrhagia OR menstru* OR menses OR menstrual OR oligomenorrhea* OR polymenorrhea OR "polycystic ovary syndrome" OR premenstrual OR "sclerocystic ovar*" OR "stein leventhal syndrome" OR tampon*) ) OR AB ( ("abnormal uterine bleeding" OR amenorrhea* OR dysmenorrhea* OR endometrioma* OR endometriosis OR "heavy period*" OR hypomenorrhea OR "irregular uterine bleeding" OR menarche OR menorrhagia OR menstru* OR menses OR menstrual OR oligomenorrhea* OR polymenorrhea OR "polycystic ovary syndrome" OR premenstrual OR "sclerocystic ovar*" OR "stein leventhal syndrome" OR tampon*) ) \| 33,105 \| \| S6 \| (MH "Polycystic Ovary Syndrome") \| 4,745 \| \| S5 \| (MH "Endometriosis") \| 5,700 \| \| S4 \| (MH "Menstrual Hygiene Products+") \| 333 \| \| S3 \| (MH "Menstruation Disorders+") \| 8,365 \| \| S2 \| (MH "Menstrual Cycle+") \| 7,465 \| \| S1 \| (MH "Menarche") \| 1,556 \| | |

1. Bramer W, Bain P. Updating search strategies for systematic reviews using EndNote. J Med Libr Assoc. 2017;105(3):285-9.

2. Clark JM, Sanders S, Carter M, Honeyman D, Cleo G, Auld Y, et al. Improving the translation of search strategies using the Polyglot Search Translator: a randomized controlled trial. J Med Libr Assoc. 2020;108(2):195-207.

3. Bramer WM, Giustini D, de Jonge GB, Holland L, Bekhuis T. De-duplication of database search results for systematic reviews in EndNote. J Med Libr Assoc. 2016;104(3):240-3.
